# Immunohistological and electron microscopy profile of unique TIRM-MRI guided muscle biopsies of FSHD patients

**DOI:** 10.1177/22143602251380426

**Published:** 2025-10-06

**Authors:** Anna Greco, Benno Kusters, Ritse Mann, Jurgen Futterer, Leon de Jong, Yordy Welling, Marieke Ploegmakers, Ger JM Pruijn, Leo AB Joosten, Baziel G. M. van Engelen

**Affiliations:** 1 Department of Neurology, 6034 Radboud University Medical Center, Nijmegen, The Netherlands; 2 Laboratory of Experimental Internal Medicine, Department of Internal Medicine, Radboud University Medical Center, Nijmegen, The Netherlands; 3 Department of Pathology, 6034 Radboud University Medical Center, Nijmegen, The Netherlands; 4 Department of Radiology, 6034 Radboud University Medical Center, Nijmegen, The Netherlands; 5 Department of Biomolecular Chemistry, Institute for Molecules and Materials, 6029 Radboud University Nijmegen, Nijmegen, the Netherlands

**Keywords:** FSHD, inflammation, MRI-guided biopsies, biomarker

## Abstract

**Background:**

FSHD is an inherited myopathy with complex epigenetic pathogenesis and no causal treatment. Inflammation is thought to contribute to muscle pathology, but its nature remains unclear.

**Objective:**

To characterize inflammatory infiltrates and morphological changes in MRI-guided FSHD muscle biopsies compared to healthy controls (HC).

**Methods:**

We performed turbo inversion recovery magnitude (TIRM) and DIXON MRI on 43 genetically confirmed FSHD patients (50 ± 12 years, 51% men) to assess inflammation and fatty infiltration. From 24 patients with at least one TIRM + leg muscle, two MRI-guided biopsies (TIRM + and TIRM−) were obtained. Needle biopsies from 8 HC (36 ± 12 years, 62% men) served as controls. Samples underwent hematoxylin-phloxine staining and immunodetection of CD3, CD4, CD8, CD56, CD68, HLA-ABC, HLA-DR, and MAC. Electron microscopy provided ultrastructural analysis.

**Results:**

TIRM + FSHD samples showed significantly higher histopathology and inflammation grades than paired TIRM− and HC samples. Inflammatory infiltrates, mainly CD8 + lymphocytes and CD68 + macrophages, were present in 67% of TIRM + and 20% of TIRM− muscles. Electron microscopy revealed frequent myofibrillar disorganization in TIRM + samples.

**Conclusion:**

Our findings validate TIRM hyperintensity as a biomarker for active disease, correlating with histopathology and inflammation. The incidence of inflammation in FSHD appears underestimated, highlighting its role in disease pathogenesis. These results support targeting inflammation as a potential therapeutic strategy in FSHD.

## Introduction

Facioscapulohumeral muscular dystrophy (FSHD) is an autosomal inherited muscle disorder.^1^ The clinical phenotype varies greatly between and within FSHD families^2^ and the classical clinical features include (1) typical onset between 15 and 30 years, (2) progressive and frequently asymmetric weakness and atrophy of the facial, shoulder, and upper arm muscles.^1^ With a prevalence of ∼1 in 8000 FSHD is actually one of the more common hereditary myopathies.^3^ The disease mechanism is complex involving both genetic and epigenetic factors.^4^ Briefly, two genotypes (FSHD1, 95% of patients, and FSHD2, less than 5%) result in the same phenotype.^5^ In the case of FSHD1, the contraction of the macroarray D4Z4 at the tip of a permissive chromosome 4 to 10 or fewer repeat units results in chromatin rearrangement with consequent expression of a gene called Double Homeobox 4 (DUX4).^6^ In the case of FSHD2, it is not the region contraction, but the mutation of other chromatin maintainers (such as but not limited to SMCHD1) that causes chromatin remodeling and DUX4 expression.^7^ According to this model, both (epi)genetic mechanisms eventually result in DUX4 protein expression, which is toxic to skeletal muscle cells.^8^ Possible DUX4 toxicity mechanisms have been and still are vividly under investigation, with inflammation being one of them.^9^ Inflammation has recently gained attention and may be seen as a side effect of the DUX4 model as well as an active contributor to muscle pathology once activated. The second perspective has clear implications as it may be one of the roads eventually leading to a causal treatment. So far, muscle inflammatory infiltrates have been described in up to one-third of muscle biopsies and are mainly composed of lymphocytes.^10–13^ In addition, the following unspecific major histology features have been observed: a great fiber size variation, abnormal central nucleation, increased number of necrotic and regenerating fibers, and increased fibrosis.^12^ In addition to (invasive) histology studies, MRI has gained predictive value to identify inflammation as well. In fact, TIRM hyperintensity on muscle MRI has been recognized as imaging biomarker of active disease in FSHD patients and included in the design of clinical trials.^14,15^ Recent longitudinal MRI studies have solidified the role of quantitative imaging as both prognostic and efficacy biomarkers. Vincenten et al.^16^ showed in 105 FSHD patients that changes in a composite MRI score (MRI-CoS), derived from fat fraction and TIRM positivity, significantly correlated with clinical severity over a 5-year period, especially in patients with moderate baseline involvement or multiple TIRM-positive muscles. Similarly, a recent study of Paoletti *et al.*^17^ applied multiparametric MRI including fat fraction and water-T_2_ (wT_2_) measurements in TIRM-positive muscles and demonstrated that muscles with intermediate fat replacement or elevated wT_2_ at baseline were more prone to progression, with imaging measures correlating with clinical scales. Together with emerging histological indicators such as fibrosis as a prognostic factor,^18^ these findings underscore a convergent multi-modal biomarker strategy centered on inflammation, structural damage, and MRI signals. However, the relationship between MRI changes, specific inflammatory cell populations, molecular immune activation, and ultrastructural muscle damage in FSHD remains incompletely characterized.

In this prospective cross-sectional study, we examined the correlation between MRI characteristics and muscle histopathology, muscle inflammation and morphological ultrastructural changes in a cohort of well-characterized FSHD patients (N = 43) alongside 8 unrelated healthy individuals taken as negative controls. Importantly, within the patient group we discriminated between muscle with and without sign of active disease (MRI TIRM status). Additionally, we explored the association between histopathology and inflammation grades and clinical indicators of disease severity. By integrating imaging, histological, immunological, and ultrastructural data, our work provides a comprehensive, multi-level analysis of muscle pathology in FSHD, offering novel insights into the interplay between inflammation and structural muscle damage and reinforcing the value of TIRM hyperintensity as a clinically relevant biomarker.

## Material and methods

### Participants

In this study we included 43 patients with FSHD and 8 unrelated healthy controls. We invited all included participants from 2019 to 2020 at the neurology outpatient clinic of Radboud University Medical Center, Nijmegen, The Netherlands. Patients with FSHD met all the following criteria: (1) genetically proven FSHD (type 1 or 2); (2) older than 18 years; (3) absence of any infectious or inflammatory conditions; (4) absence of any autoimmune disorder (for instance Graves’ disease, Hashimoto thyroiditis, multiple sclerosis, myasthenia gravis, Pernicious anemia, Addison's disease, celiac disease, dermatomyositis, inclusion body myositis, rheumatoid arthritis, autoimmune vasculitis, type 1 diabetes, etc); (3) past or present history of malignancy; (4) use of corticosteroids; (5) use of statins; (6) use of anti-inflammatory medication. Unrelated healthy controls fulfilled the following criteria: (1) older than 18 years; (2) absence of any personal/family medical history of neuromuscular disorders; (3) normal muscle strength during physical examination; (4) absence of any issues listed from point 3 to 6, as for the patient group.

We recorded for both patients and controls demographics (sex, age at examination, race) and anthropometrics (height, body mass index). Additionally, we assessed for the patient group the following clinical features: disease duration, disease severity scores by means of the Ricci (0–10 point severity scale^19^) and Lamperti score (0–15 point severity scale^20^), Medical Research Council sum score (MRC Sum Score) ranging from 0 (paralysis) to 60 (normal muscle strength), FSHD type and D4Z4 repeat length. Additionally, the following scores were obtained: Sit Up Score (patient is asked to sit up with his/her arms across chest while knees are bent and the examiner hold patient's ankles, the test is scored as 0, able to fully perform task, or 1, only able to lift head); Supine to Sit Score (patient is in supine position, arms at his/her side on the examination bench while examiner hold his/her ankles and ask patient to sit up as fast as he/she can; the score ranges from 0 (able to perform task within 3 s) to 4 (unable); Timed Up and Go (TUGO), which is the time (in seconds) needed to stand up from a chair, walk a distance of 3 meters, turn, come back, and sit again; the score ranges from 0 (able to perform task within 6 s) to 4 (unable); 10 Meter Walk/Run test (10MWR) where patient is asked to traverse 10 meters as fast as possible, the score ranges from 0 (able to perform task within 4 s) to 4 (unable) and maximum voluntary contraction of tibialis anterior (MVC TA).^21^ Additionally, total fat and muscle mass were obtained from FSHD patients through a bioelectric impedance analysis (BIA) using Tanita BC 601 (Tanita corporation, Tokyo, Japan). Participants’ characteristics are summarized in Table 1.

**Table 1.** Subjects characteristics.

	FSHD Patients	Healthy Controls
*Demographics & Antropometrics*		
Number of participants (% male)	43 (51%)	8 (62%)
Age at examination mean y** **±** **SD	50 ± 12	36 ± 12
BMI mean** **±** **SD	25 ± 3	24 ± 2
Asian background n/tot. n	1/43	0
Caucasian background n/ tot. n	42/43	8/8
*Clinical outcomes*		
Disease duration mean y** **±** **SD	24 ± 16	n/a
MRC Sum Score (0–60) mean** **±** **SD	53 ± 6	60
Ricci Score (01–10) mean** **±** **SD	5 ± 2	n/a
Lamperti Score (0–15) mean** **±** **SD	6 ± 3	n/a
Sit Up Sore (0–1)		
0 (able)	15/43	n/a
1 (unable)	28/43	n/a
Supine to Sit Score (0–4)		
➣0 (< 3 s)	17/43	n/a
➣ 1 (3.1–6 s)	6/43	n/a
➣ 2 (6.1–9 s)	6/43	n/a
➣ 3 (> 9.1 s)	6/43	n/a
➣ 4 (unable)	8/43	n/a
*TUGO (0–4)*		
➣ 0 (< 6 s)	2/43	n/a
➣ 1 (6.1–8 s)	18/43	n/a
➣ 2 (8.1–10 s)	9/43	n/a
➣ 3 (10.1–12 s)	11/43	n/a
➣ 4 (> 12 s)	3/43	n/a
*10 MWR (0–4)*		
➣ 0 (< 4 s)	1/43	n/a
➣ 1 (4.1–8 s)	28/43	n/a
➣ 2 (8.1–12 s)	10/43	n/a
➣ 3 (> 12 s)	4/43	n/a
➣ 4 (unable)	0	n/a
*BIA*		
Fat mass (%)	31 ± 10	n/a
Muscle mass (kg)	50 ± 17	n/a
*MRI status*		
TIRM -	19/43	n/a
TIRM +	24/43	n/a
*Genetics*		
FSHD1 n/tot. n	42/43	n/a
FSHD2 n/tot. n	1/43	n/a
D4Z4 repeat units mean** **±** **SD	7 ± 2	n/a

The study was performed after approval by the local medical ethical committee (CMO Arnhem-Nijmegen) under file number NL64690.091.18 and in accordance with Good Clinical Practice guidelines. All participants provided written informed consent.

### MRI protocol and MRI guided biopsies

To study the differential histological features of inflamed (presumably active disease) and not inflamed FSHD muscles we collected highly targeted MRI-guided biopsies. First, all FSHD patients included in the study underwent a screening whole-leg MRI examination as detailed previously by our group.^22,23^ To this end, we obtained transversal Dixon and TIRM sequences from all patients to assess muscle fatty infiltration and inflammation, respectively (for detailed MRI protocol description, refer to the Supplemental Methods of our recent study^23^). Next, based on this first screening MRI, 24 patients out of 43 showed at least one TIRM-positive muscle leg and underwent within 6 weeks a second targeted MRI scan to confirm the consistent presence of TIRM hyperintensity in the selected muscle, followed immediately by the MRI-guided biopsy procedure as detailed previously by our research group.^24^ Briefly, we collected two targeted muscle samples from each selected patient: (1) one TIRM-negative, healthy-appearing muscle (23/24 vastus lateralis), and (2) a second sample targeting a TIRM-hyperintense muscle (15/24 gastrocnemius medialis), presumably in the active disease phase. The included unrelated healthy controls (n = 8) did not undergo an MRI-guided biopsy but a Bergström needle muscle biopsy from the vastus lateralis muscle.^25^

### MRI qualitative and quantitative analysis

Inflammation on MRI was qualitatively scored as either TIRM-positive (hyperintensity on MRI) or TIRM-negative (normal intensity on MRI) by an experienced radiologist.

The degree of muscle fatty infiltration was quantitatively analyzed for 36 out of the 43 FSHD patients as described in our recent previous work.^26^ Briefly, we calculated a fat fraction map based on the reconstructed water and fat images signal of the DIXON sequences applying in MATLAB (version R2014B, The Mathworks, Inc. Natick, Massachusetts, United States), using the following equation:

$$FF=\frac{F}{F+W}$$

Based on the formula, we divided the signal intensity of the fat image (F) by the signal intensities of the fat and water (W) images together thereby obtaining the fat fraction (FF). After obtaining the fat fraction map, we manually outlined the contours of 12 upper leg muscles (sartorius, gracilis, vastus medialis, vastus lateralis, vastus intermedius, rectus femoris, biceps femoris caput brevis, biceps femoris caput longus, semitendinosus, semimembranosus, adductor magnus, and adductor longus) and of 7 lower leg muscles (tibialis anterior, extensor digitorum longus, peroneus, tibialis posterior, soleus, gastrocnemius medialis, and gastrocnemius lateralis) at specific regions of interest as previously described by Mul et al.^22^ Next, we calculated the average fat fraction within each muscle contour ( ${\bar{FF}}_{i}$ ) using ImageJ software. Finally, we calculated per patient a comprehensive average lower limb fat fraction (LLFF) weighted on the area (A) of each muscle following this equation:

$$LLFF=\frac{\sum_{i=1}^{n}\;A_{i}\cdot {\bar{FF}}_{i}}{\sum_{i=1}^{n}\;A_{i}},\;n:\;\mathrm{muscle}\;\mathrm{contours}\;\mathrm{of}\;\mathrm{one}\;\;\mathrm{patient}$$

### Histological and immunostaining analysis

After collection, muscle biopsies were immediately snap frozen in liquid nitrogen-cooled isopentane and stored at −80 ˚C. Serial muscle cryosections of 6 µm were obtained from each sample using using Leica CM3050S cryostat at −23°C. Cryosections were stained with hematoxylin-phloxine (Hphlox) to evaluate: 1) variability in fiber size, 2) extent of central nucleation, 3) presence of necrosis/regeneration, 4) presence of interstitial fibrosis. The severity of each category was scored as normal (0), mild (1), moderate (2), or severe (3) as previously described by Lassche et al.^27^ Briefly, the sum score of each category was used to assess the pathology grade, which ranges from 0 (normal) to 12 (severe muscular dystrophy). In addition, inflammation was graded separately from 0 to 3 based on the extent of abnormalities as previously described.^27^ In addition, immunohistochemistry was performed on serial muscle cryosections using the following primary antibodies (clone number; firma): CD3 (5740; Dako), CD4 (SP35; Cell Marque), CD8 (C8-144B; Dako), CD56 (123C3; Dako), CD68 (KP1; Dako), HLA-ABC (W6-32; Exbio), HLA-DR (TAL-1B5; BD), and MAC (aE11; Dako). Pathology and inflammation grade as well as microscopic evaluation of CD3, CD4, CD8, CD56, CD68, HLA-ABC, HLA-DR, and MAC staining were evaluated by an experienced neuropathologist unaware of the exact diagnosis of each sample.

### Correlation of histopathology and inflammation grade with muscle derived IL-6

We refer to the methods section of our recently published study for a detailed description of ex vivo muscle stimulation procedures and IL-6 measurement.^23^ Briefly, we stimulated for 24 hours TIRM-positive and TIRM-negative muscle samples of 21 out of 24 FSHD patients with the following stimuli: culture medium as negative control (DMEM), LPS, and Pam3Cys-Ser-(Lys)4 (P3C). Following up on our previous results,^23^ in this study we investigated whether the obtained histopathology and inflammation grades were associated with the concentration of IL-6 measured in the supernatant of ex vivo-stimulated muscle biopsies.

### Electron microscopy analysis

Immediately after collection, muscle samples were fixed in 2% glutaraldehyde buffered with 0.1 M sodium-cacodylate, pH 7.4, and postfixed in 0.5% potassium hexacyanoferrat (II)-trihydrate in 1% osmium tetroxide in Palade buffer, dehydrated in ethanol and propylene oxide, and embedded in Epon. Ultrathin sections (80 nm) from a subset of randomly selected muscle biopsies of 15 FSHD patients (TIRM + as well as TIRM- per each sample) and 5 healthy controls were sliced using the UC6 Leica Ultramicrotom and examined using the JEOL JEM-1400 electron microscope (80 kV or 120 kV). There is no standardized electron microscopy scoring system available for FSHD. We scored the degree of damage of myofibrils and mitochondria from 0 (normal) to 2 (severe changes).

### Statistical analysis

All statistical analyses were conducted using GraphPad prism 5 (GraphPad software, La Jolla, CA) or SPSS version 21.0 (SPSS Inc, Chicago, IL). Means were compared with the non-parametric Mann Whitney test. Results were expressed as mean ± SD. Spearman rho analyses were used to determine correlations. Differences with a 2-sided *p* level < 0.05 were considered significantly different

## Results

### Participants

In this study 43 patients with FSHD (aged 50 ± 12 years, 51% men) and 8 unrelated healthy controls (aged 36 ± 12 years, 62% men) were included. Demographic, clinical, and genetic characteristics of participants are listed in Table 1. Full clinical annotations of included patients and controls are provided in Supplementary Table 1.

### Muscle biopsies selection

Twenty four FSHD patients out of 43 showed at least one TIRM hyperintense (TIRM+) muscle and were therefore selected to undergo the MRI-guided biopsy procedure, consisting of collecting one TIRM- and one TIRM + muscle biopsy per patient. The most frequently normal-appearing muscle with no signs of inflammation was the vastus lateralis, while the most frequently inflamed muscle was the gastrocnemius medialis. Therefore, we collected 23 out of 24 vastus lateralis TIRM- biopsies and 1 out of 24 vastus intermedius TIRM- biopsy. For the TIRM + biopsies, we collected 15 out of 24 gastrocnemius medialis biopsies, 1 out of 24 gastrocnemius lateralis biopsies, 2 out of 24 extensor digitorum longus biopsies, 1 out of 24 vastus lateralis biopsy, 1 out of 24 sartorius biopsy, 1 out of 24 soleus biopsy, 1 rectus femoris biopsy, and 2 out of 24 tibialis anterior biopsies.

### The degree of muscle fatty infiltration correlates with histology and inflammation grade of TIRM- FSHD muscle samples

**Figure 1.**
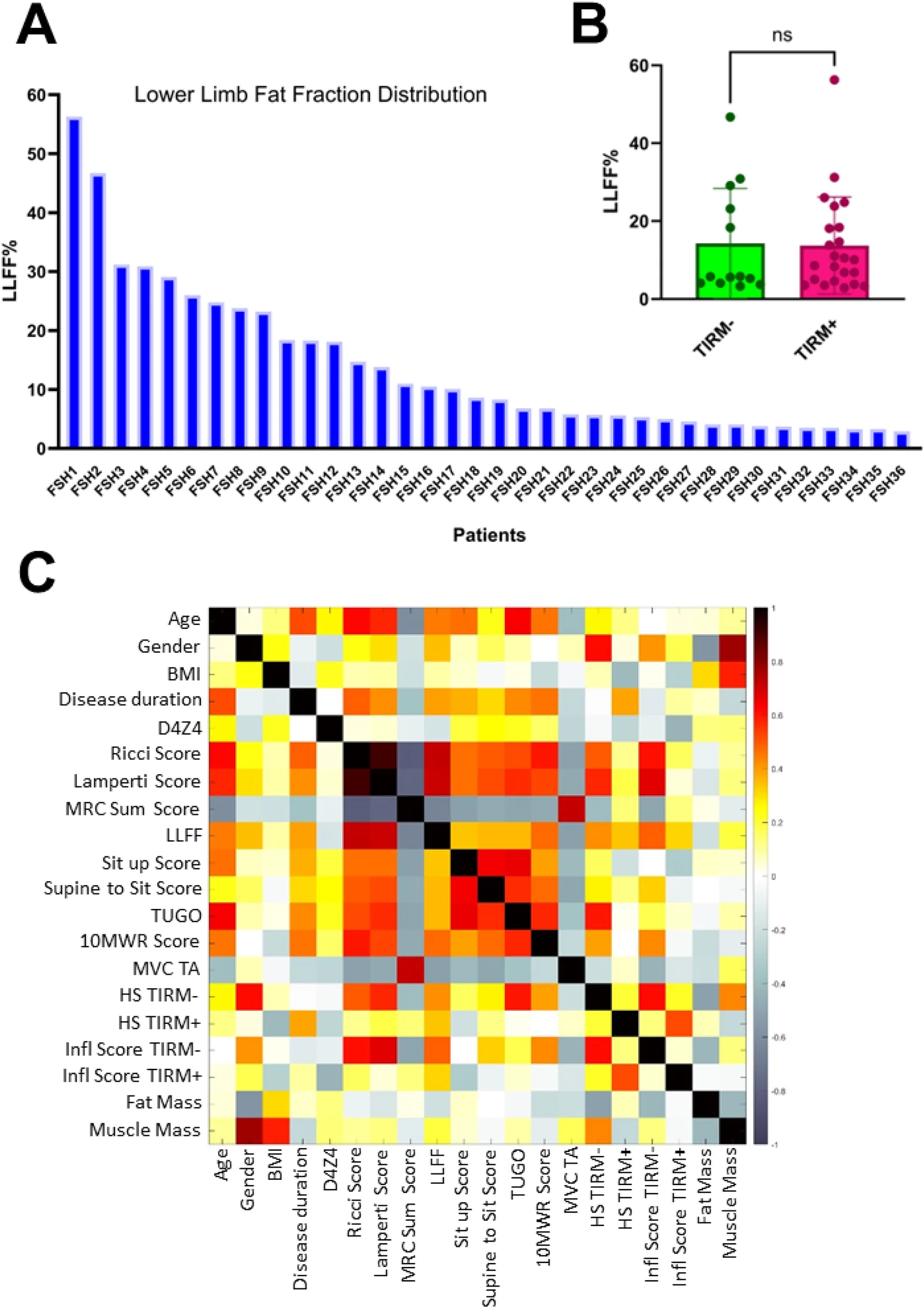
Patient cohort: degree of fatty muscle infiltration in FSHD patients and clinical correlations. (**A**) Distribution of Lower Limb Fat Fraction (LLFF) among FSHD patients (n = 36) included in the study. (**B**) Mean ± SD of LLFF between FSHD patients with TIRM normal intensity on MRI (TIRM-) and FSHD patients with TIRM hyperintensity on MRI (TIRM+). (**C**) Plot of correlogram showing pairwise Spearman correlations among 20 variables. Correlation is significant at the 0.05 level (2-tailed).

**Figure 2.**
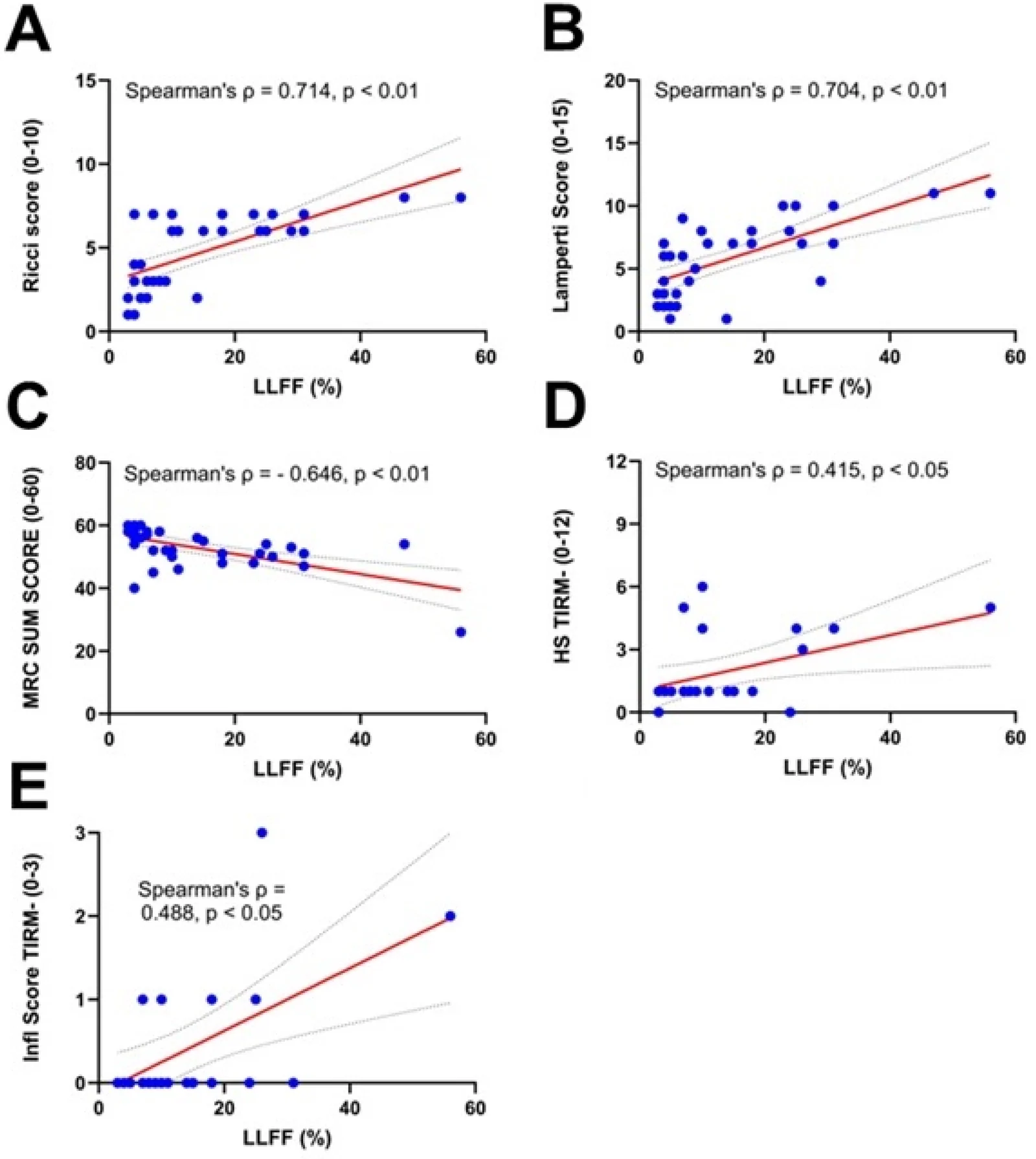
Association between degree of fatty muscle infiltration and severity, histopathology and inflammation scores. LLFF = Lower Limb Fat Fraction. HS = histopathology sum score. Infl = inflammation. Scatter dot plots showing (**A**) Ricci score, (**B**) Lamperti score, (**C**) MRC Sum Score, (**D**) Histopathology sum score of TIRM- muscle, and (**E**) Inflammation score of TIRM- muscle against LLFF.

Thirty six patient whole leg MRIs out of 43 underwent a quantitative analysis as well to extrapolate the degree of muscle fatty infiltration, provided as LLFF, as described in the methods section. The distribution of LLFF among the patient group is shown in Figure 1-A, ranging from 2.9% to 56.3%. Additionally, we observed that LLFF had a strong correlation (coefficient value between ± 0.50 and ± 1) with disease severity (Ricci and Lamperti Score), and muscle weakness (MRC Sum Score) (Figure 1-C and Figure 2A–C)). Moreover, LLFF had a moderate correlation (coefficient value between ± 0.30 and ± 0.49) with the histopathology and inflammation scores of the TIRM- FSHD muscle samples (Figure 1-C and Figure 2D–E)) but not with the corresponding TIRM + samples. The full correlations table including all correlation coefficients and corresponding p-values is provided as Supplementary Table 2. Supplementary Table 3 contains all results of the quantitative MRI analysis, providing the single fat fraction of each muscle section and the area of each muscle.

### TIRM+ muscle samples have a higher histopathology and inflammation score than paired TIRM- muscles samples and healthy control samples

**Figure 3.**
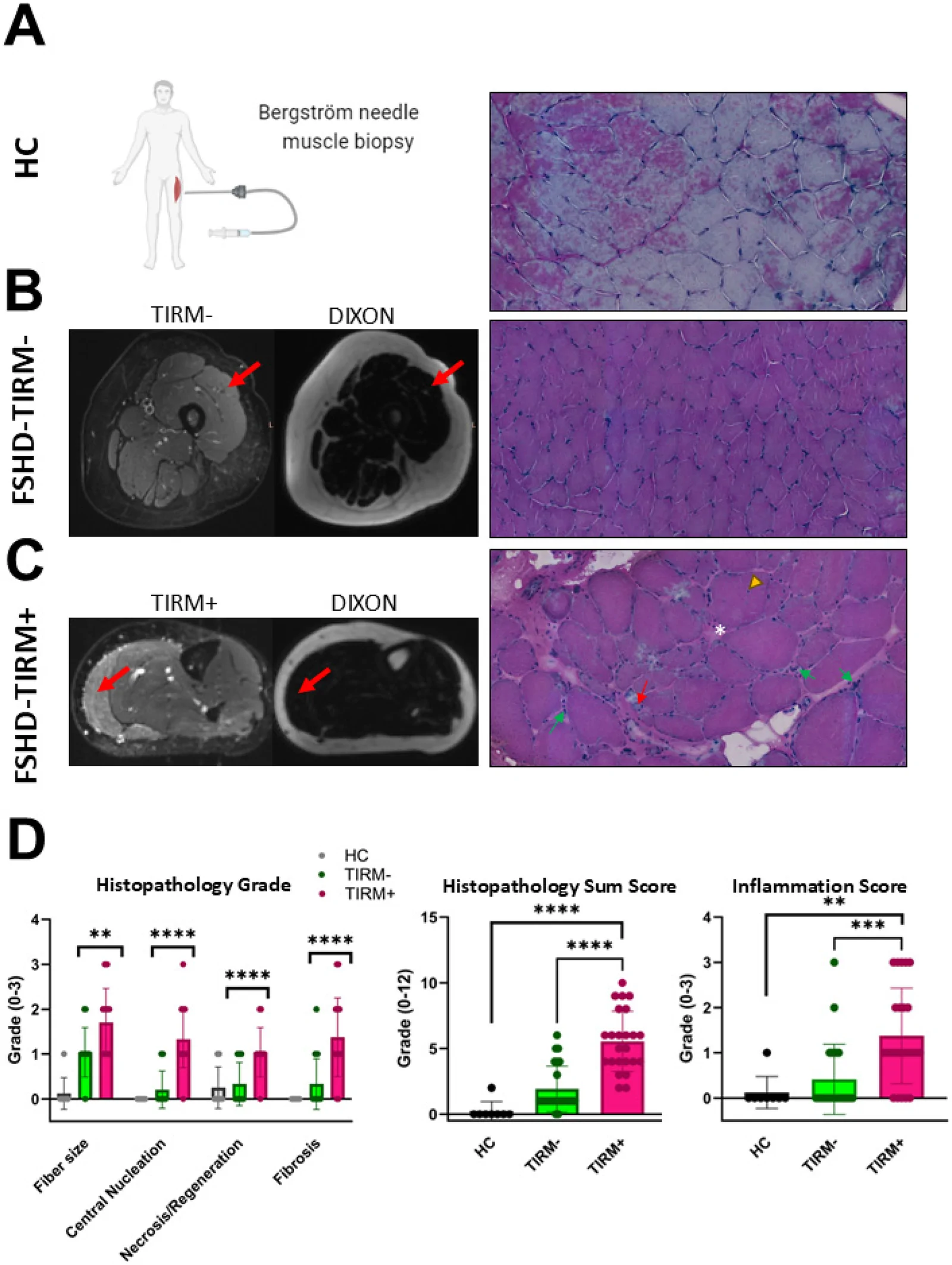
Histopathology and inflammation grade of MRI guided FSHD muscle biopsies. Bergstrom needle vastus lateralis muscle biopsies were collected from 8 healthy controls. MRI guided muscle biopsies targeting a normal appearing vastus lateralis muscle with no sign of inflammation (TIRM-) were collected from 24 FSHD patients alongside with a paired biopsy collected from a TIRM + positive muscle (presumed inflammation). (**A**) needle vastus lateralis muscle biopsy of a representative healthy control and corresponding H-phlox staining showing normal histology and no inflammation. (**B**) TIRM- MRI guided vastus lateralis muscle biopsy of a representative FSHD patient showing normal findings and no inflammation. Arrows indicate the site of the biopsy. (**C**) TIRM + MRI guided gastrocnemius muscle biopsy of the same FSHD patient and corresponding HPhlox staining showing fiber size variation, internal nucleation (yellow arrowhead), fibrosis (white asterisk), a few regenerating muscle fibers (red arrow) and endomysial inflammatory cells (green arrows0. (**D**) From left to right: 1) Mean ± SD of histopathology sub scores (ranging from 0, normal, to 3, severe) of healthy controls muscle biopsies, FSHD-TIRM- muscle biopsies, and FSHD-TIRM + muscle biopsies; 2) Mean ± SD of histopathology sum scores (each ranging from 0, normal finding, tot 12, severe dystrophic changes) of controls muscle biopsies, FSHD-TIRM- muscle biopsies, and FSHD-TIRM + muscle biopsies; 3) Mean ± SD of inflammation scores (ranging from 0, normal, to 3, severe) of controls muscle biopsies, FSHD-TIRM- muscle biopsies, and FSHD-TIRM + muscle biopsies. ** p < 0.01; ***; p < 0.001 p < 0.0001.

FSHD-TIRM+ muscle samples had more severe variation in fiber size, presence of internal nucleation, degree of necrosis/regeneration, and of fibrosis (Figure 3-C and D). The mean histopathology sum score (ranging from 0 to 12) of FSHD-TIRM+ muscle samples was 5.5 ± 2.3, significantly higher compared to those of the corresponding FSHD-TIRM- samples (1.9 ± 1.7) and of healthy control muscle samples (0.3 ± 0.7). Similarly, the mean inflammation score of the FSHD-TIRM+ muscle samples (1.4 ± 1.1) was significantly higher than that of FSHD-TIRM- samples (0.4 ± 0.8) and of healthy control muscle samples (0.1 ± 0.3) (Figure 3-D).

### The histopathology grades of TIRM- muscle samples correlate with the muscle IL-6 production capacity

After having assessed the histopathology and inflammation grade of TIRM- and TIRM + FSHD muscle samples, we investigated the association with muscle derived IL-6. We found that the histopathology grade of TIRM- FSHD muscle samples had a mild correlation with the concentration of IL-6 produced by the same samples after ex vivo stimulation. We did not observe a correlation between the histopathology grade of FSHD-TIRM + muscle samples and the corresponding IL-6 production. Correlation coefficients are summarised in Table 2.

**Table 2.** Overview of Spearman correlation between histopathology and inflammation grade with muscle derived IL-6.

Variable		TIRM- mIL6 DMEM	TIRM- mIL6 LPS	TIRM- mIL6 P3C
HS TIRM-	**r_S_**	0.475*	0.501*	0.540*
Infl Score TIRM-	**r_S_**	−0.090	0.138	0.425

Variable		TIRM + mIL6 DMEM	TIRM + mIL6 LPS	TIRM + mIL6 P3C
HS TIRM+	**r_S_**	0.097	0.421	0.299
Infl Score TIRM+	**r_S_**	−0.173	−0.183	−0.189

### Inflammation on MRI correlates with inflammation on immunohistochemical level

**Figure 4.**
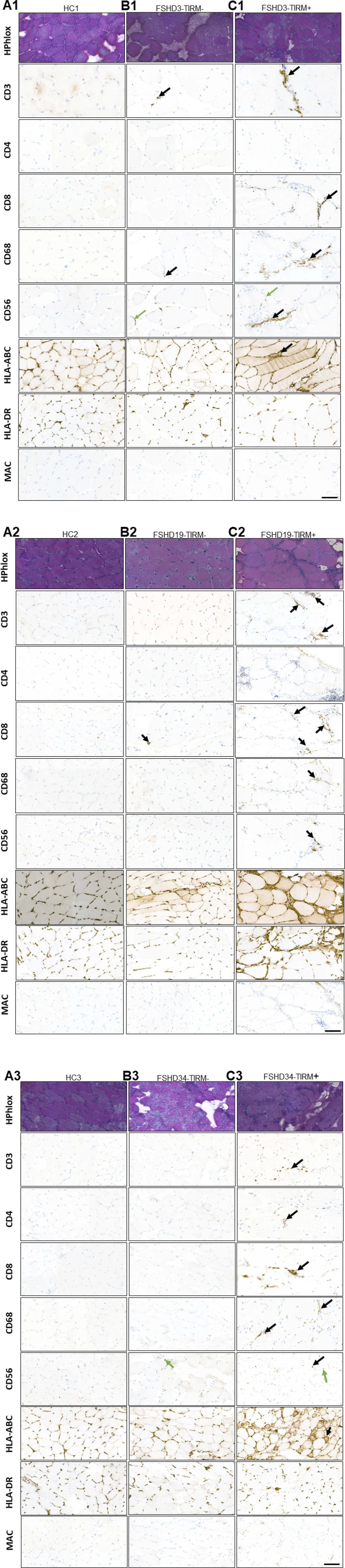
Histological and immunohistochemical analyses of FSHD MRI guided muscle biopsies and needle healthy muscle biopsies. Images for 3 healthy controls and 3 FSHD patients are shown. Representative HPhlox and immunostaining (CD3, CD4, CD8, CD56, CD56, HLA-ABC, HLA-DR, and MAC, respectively) results of serial cryo-sections from needle muscle biopsies of 3 healthy controls (A1-A3: HC1, HC2, and HC3, respectively), and 3 patients (FSHD3, FSHD19, and FSHD34). B1-B3: TIRM- muscles of patients FSHD3, FSHD19, and FSHD34 respectively; C1-C3: TIRM + muscles of patients FSHD3, FSHD19, and FSHD34 respectively. Arrows point at the presence of positive staining of the corresponding marker indicated in each panel. In case of CD56 panel, black arrows point at round-shaped cells expressing CD56 and green arrows point at elongated-shaped cells at the direct periphery of myofibers. Scale bars in all pictures, 50 μm.

Consistent with the inflammation scores previously reported, we found inflammatory infiltrates in 16/24 FSHD-TIRM + muscle samples, 5/24 FSHD-TIRM- muscle samples, and one micro-infiltrate in 1/8 HC. Sporadically, a few cells not organized in clusters were observed in all three types of muscle samples (healthy, TIRM-, and TIRM+). This is a normal finding as leukocytes can reside in muscle as well.^28^ Immunohistochemical images for 3 representative patients and 3 healthy controls are provided in Figure 4. The TIRM + muscle samples have clear infiltrates mainly composed of CD3 + CD8 + lymphocytes and of macrophages (CD68+). Sporadic cells expressing the marker CD56 and located at the direct periphery of the myofibers were appreciated in all three types of muscle samples (healthy, TIMR-, and TIRM+). However, CD56-expressing cells were more prominent in the FSHD-TIRM + muscle samples, where based on morphology and localization, we observed two types of cells. Elongated-shaped cells at the direct periphery of myofibers with CD56-positive staining around the nucleus: these cells, based on morphology, localization, and CD56-positive staining, can be interpreted as satellite cells (patient C3, Figure 3). However, we also observed round-shaped cells expressing CD56, which are not located at the direct periphery of the myofibers but in the endomysial space. These cells may be interpreted as leukocytes, and possibly as NK cells (patients C1 and C2, Figure 4 ). Additionally, prominent HLA-ABC upregulation was observed in the FSHD-TIRM + positive samples, ranging from patchy sarcolemmal and/or cytoplasmic patterns to a diffuse severe pattern. We did not observe severe diffuse HLA-ABC upregulation in the FSHD-TIRM- muscle samples. Furthermore, we did not observe HLA-DR or MAC upregulation in any of the samples.

### Electron microscopy analysis shows myofibrillar disorganization in the FSHD-TIRM + samples

**Figure 5.**
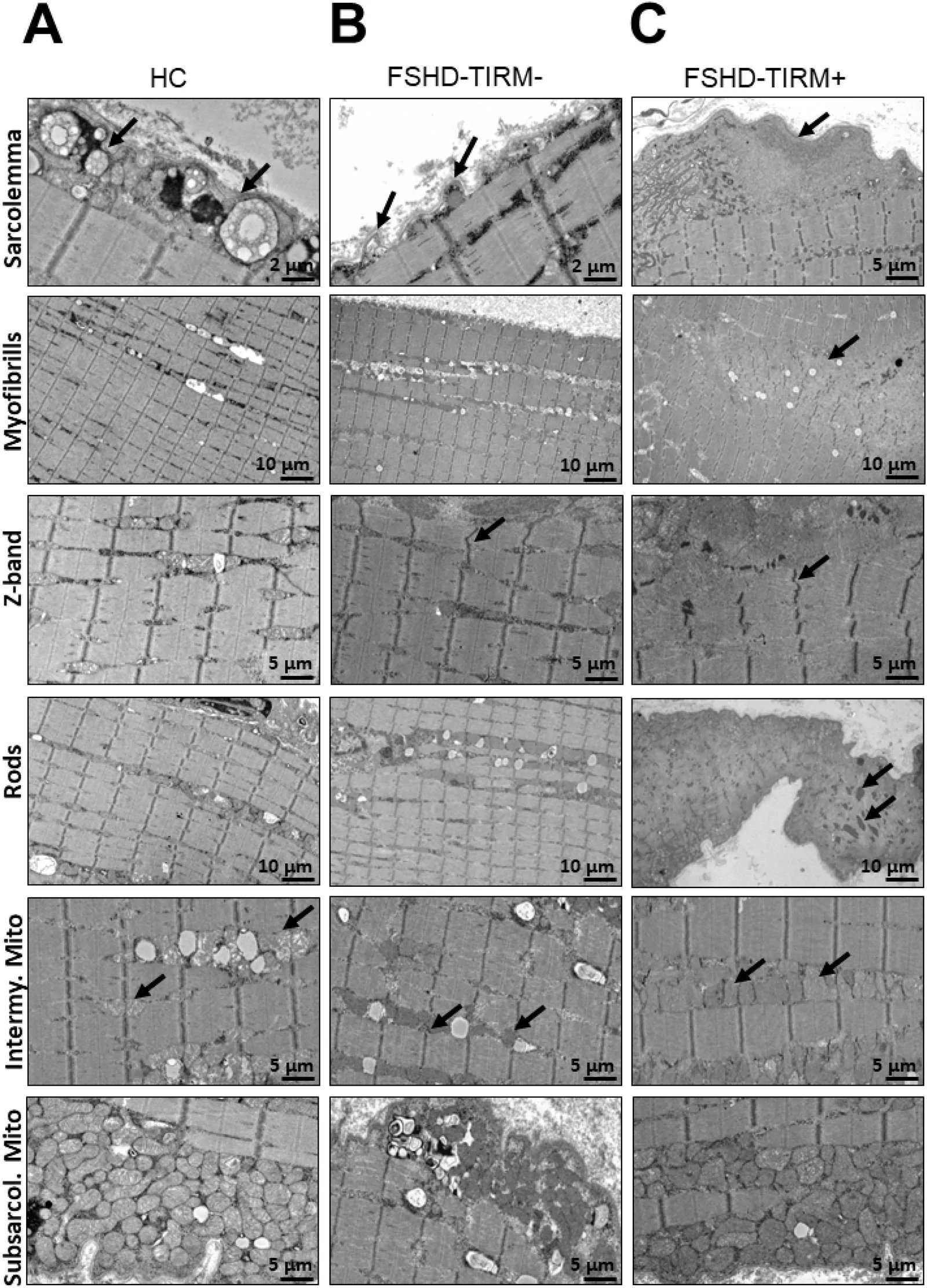
Morphological ultrastructure of muscle tissue visualized by electron microscopy. (A) Muscle needle biopsies of healthy controls, (B) TIRM- MRI guided muscle biopsies of FSHD patients and (C) TIRM + MRI guided muscle biopsies of the same FSHD patients. Representative data obtained with material from a single control and a single patient are shown. Arrows indicate the structure named on the left (sarcolemma, myofibrils, Z-band, rods, intermyofibrillar and subsarcolemmal mitochondria).

**Figure 6.**
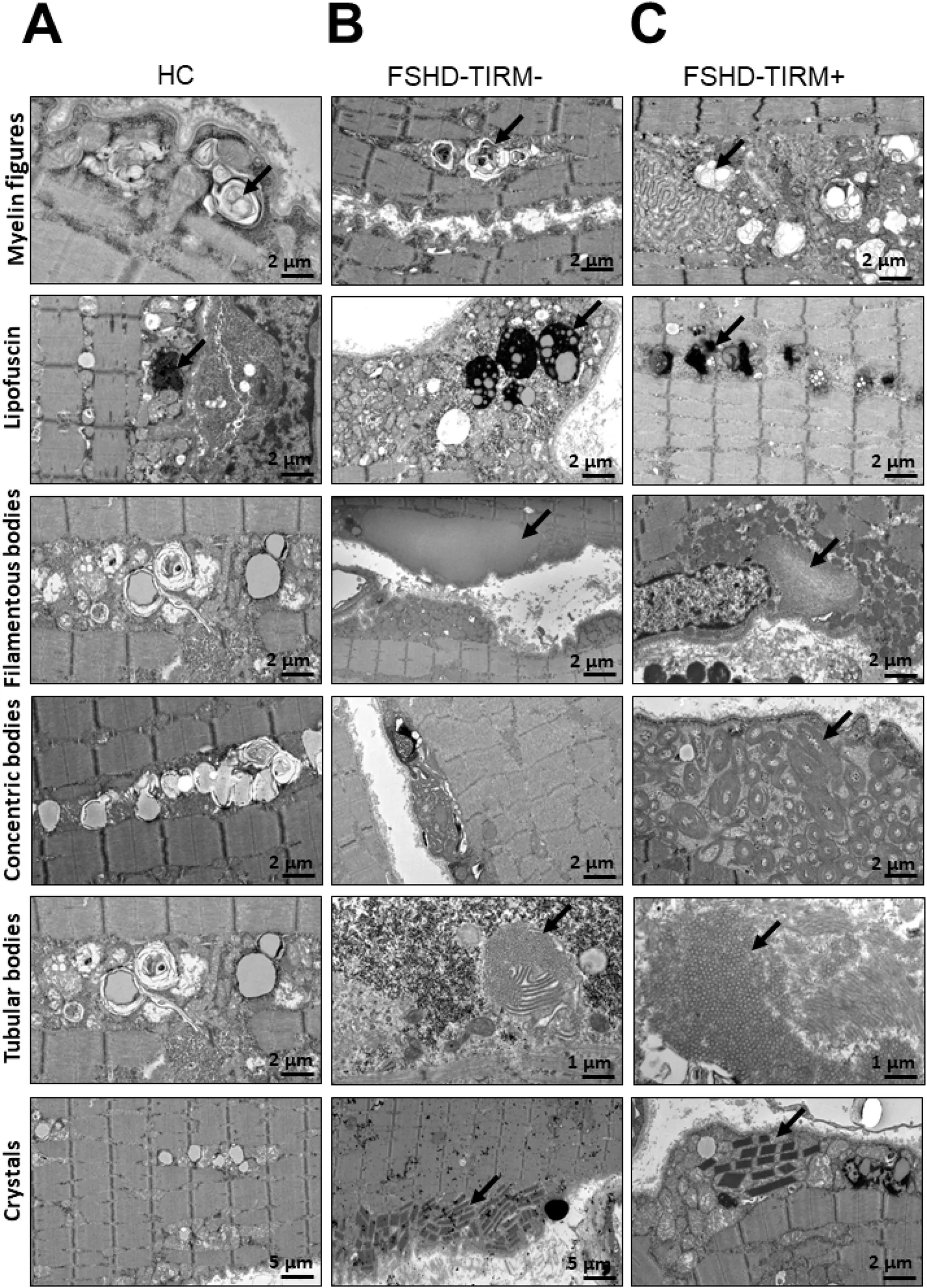
Inclusions in muscle tissue visualized by electron microscopy. A) Muscle needle biopsies of healthy controls, (B) TIRM- MRI guided muscle biopsies of FSHD patients and (C) TIRM + MRI guided muscle biopsies of the same FSHD patients. Representative data obtained with material from a single control and a single patient are shown. Arrows indicate the structure named on the left (myelin figures, lipofuscin, filamentous bodies, concentric bodies, tubular bodies, crystals).

Finally, in order to identify morphological changes possibly associated with inflammation (TIRM hyperintensity), we provided a qualitative descriptive analysis of the ultrastructural features characterizing muscle biopsies of paired TIRM + and TIRM- muscle samples (n = 15 FSHD patients) alongside of healthy control muscle samples (n = 5). However, except for more frequent myofibrillar disorganization, no other specific alterations were found in the TIRM + muscle samples. Generally, we observed that the sarcolemma of control muscle specimens (Figure 5-A) firmly adhered to subsarcolemmal structures (such as lipid droplets and mitochondria) closely following their contours. Conversely, in the patient group and more often in the TIRM + samples (Figure 5B–C), we observed aspecific alteration of the sarcolemma, which had a more loose profile not closely following the contour of subsarcolemmal structures, especially in areas with more pronounced myofibrillar disorganization (Figure 5C). FSHD-TIRM- muscle samples had a normal myofribrillar organization comparable to that of healthy control muscle samples (Figure 5A–B). Conversely, in the FSHD-TIRM + muscle samples, it is possible to appreciate focal areas of disorganization (cores), alongside streaming of the Z-bands and rod-like formations (Figure 5C). We observed intermyofibrillar mitochondria as well as conglomerates of subsarcolemmal mitochondria with significant size variation in patient (both TIRM- and TIRM+) as well as healthy control muscle biopsies, without specific alterations. Additionally, several inclusion structures were observed (Figure 6). Myelin figures (concentric lamellations mostly made up of phospholipids and calcium) and lipofuscin (granules containing lipid and protein material) were regularly observed in healthy as well as patient muscle samples and without difference between TIRM- and TIRM + samples. Filamentous bodies, tubular bodies and crystals were occasionally observed in TIRM- as well as TIRM + patient samples but in none of the healthy controls. Only in 1 out of 24 TIRM + muscle biopsies (and none of the TIRM- and healthy control muscle samples) we observed the presence of concentric bodies.

A representative overview of muscle morphological ultrastructure and inclusion structures is provided in Figures 5 and 6, respectively.

## Discussion

In this study, we provide an immunohistological and morphological analysis of MRI guided TIRM + and TIRM- muscle biopsies of well characterized (genetically, clinically and radiologically) FSHD patients and of needle muscle biopsies of unrelated healthy individuals taken as negative controls. Briefly, we observed the following main findings: (1) a more severe histopathology and inflammation grade in the TIRM + FSHD muscle samples, (2) the presence in the TIRM + FSHD muscle samples of inflammatory infiltrates predominantly containing CD8 lymphocytes and CD68 macrophages, and (3) the presence in the TIRM + FSHD muscle samples of myofibrillar disorganization.

We included a very well characterized cohort of FSHD patients (see Table 1 for detailed overview of patients characteristics). Among patient characteristics, we observed that LLFF (that is the overall muscle fatty infiltration based on whole leg MRI quantitative analysis*)* correlated with disease severity (Ricci and Lamperti Score) and with muscle weakness (MRS Scum Score) suggesting that the more fatty content the less functional muscle mass left with obvious consequences on patient clinical performance. Interestingly, LLFF correlated with the histopathology and inflammation grade of FSHD-TIRM- muscle samples, but not with those of FSHD-TIRM + muscle samples. A possible explanation might be that in FSHD muscle with MRI sign of active disease (TIRM hyperintensity) inflammation may be seen as a primary independent process and not as a mere consequence of muscle fatty infiltration, thereby explaining the lack of association between LLFF and histopathology and inflammatory changes, which may occur independently from the presence or absence of muscle fatty infiltration. Also, this explanation is supported by previous evidence. In fact, den Heuvel et al.^29^ observed in a separate and independent FSHD cohort a correlation between higher histopathology grade and FSHD muscle biopsies with increased fat content but not with TIRM hyperintensity.

As expected, and in line with previous studies,^14,29,30^ we observed that TIRM hyperintensity was associated with higher histopathology and inflammation grade validating TIRM hyperintensity as imaging biomarker of active disease and thereby highlighting its implementation in clinical trial as a non-invasive tool to select patients as well as monitor a specific treatment response. Confirming previous results,^11,13,31^ we also found in our cohort endomysial and perivascular muscle inflammatory infiltrates which are predominantly composed of CD8 lymphocytes and CD68 macrophages, consistent with an ongoing inflammatory process in FSHD muscle tissue. These infiltrates were more frequently found in the FSHD-TIRM + muscles samples. The presence of these inflammatory cells suggests an active immune response within the affected muscles, potentially contributing to the pathogenesis of FSHD. In addition, and consistent with previous findings,^13,32^ we found a prominent upregulation of HLA-ABC in the TIRM + FSHD muscle samples which may be interlinked with the presence of CD8 + lymphocytes infiltrates. HLA-ABC is a member of the Major Histocompatibility complex (MHC) system and it is specifically involved in antigen presentation to CD8 lymphocytes.^33^ In fact, also Frisullo et al.^13^ found CD8 endomysial infiltrates with increased HLA-ABC expression in FSHD inflamed muscle samples. Also, DUX4 activates in FSHD skeletal muscles hundreds of downstream genes, among which immune genes and cancer testis antigens, and therefore the upregulation of HLA-ABC may be interpreted as a consequence of the induced immune response. Recently, Hubregtse et al., found HLA-DR upregulation in 4/25 FSHD muscle samples and prominent MAC upregulation in 8/24 samples. In contrast to these findings, we did not find HLA-DR and MAC upregulation in any of our samples.

Sporadically, CD3 + CD8 + and CD68 + cells not organized in a cluster were observed in healthy control muscles as well. This is a normal finding as leukocytes normally reside also within intramuscular sites.^28^ Their immediate activation in response to injury is essential for promoting repair and regeneration.

It is important to consider whether the histological findings reported here are unique to FSHD or if they are also observed in other hereditary myopathies with inflammatory components. In several other inherited muscle diseases, such as limb-girdle muscular dystrophies (LGMD) and certain congenital myopathies, inflammatory infiltration dominated by CD8+ T cells and macrophages has been documented and is thought to contribute to muscle damage and disease progression.^34^ For example, in LGMD type 2B (dysferlinopathy), CD8 + cell infiltration and HLA-ABC upregulation have also been described.^35^While the link between inflammation and muscle fiber damage appears to be a common feature across several myopathies,^36^ the underlying triggers, such as the aberrant DUX4 expression in FSHD, may be disease-specific. Moreover, the patterns and characteristics of immune cell infiltration can vary in detail, potentially allowing for identification of disease-specific biomarkers and therapeutic targets. Comparative studies exploring similarities and differences in inflammatory responses between FSHD and other hereditary myopathies would be valuable to better understand shared and unique pathogenic mechanisms, ultimately guiding more targeted treatment strategies.

Our study also sheds light on the association between histopathology grade and inflammation in FSHD muscle tissue. We found a mild correlation between the histopathology grade of TIRM- muscle samples and the concentration of IL-6 produced by the same samples after ex vivo stimulation, suggesting a link between muscle pathology and the inflammatory response. However, we did not observe a similar correlation in TIRM + muscle samples, indicating that the inflammatory milieu in these muscles may be influenced by additional factors beyond the histopathological changes assessed in this study.

Finally, our electron microscopy analysis revealed frequent myofibrillar disorganization in the FSHD-TIRM + muscle samples, indicating structural abnormalities at the ultrastructural level. While the exact mechanism underlying myofibrillar disorganization in FSHD remains to be elucidated, it may reflect the downstream consequences of inflammation and DUX4 expression on muscle integrity. This finding adds to the growing body of evidence suggesting that inflammation may be associated with structural damage and disruption of muscle fibers in FSHD. Also, we may speculate that myofibrillar disorganization may impair muscle function and contribute to the progressive weakness and atrophy observed in FSHD patients.

Limitations of our study include: (1) the cross-sectional design, which preclude causal inferences and longitudinal assessments of disease progression and (2) the limited selection of immunomarkers used, which preclude the possibility to fully differentiate specific cell subpopulations. Future research should employ longitudinal study designs to validate our findings and investigate the dynamic changes in inflammation over time.

Overall, our findings highlight the importance of inflammation in the pathogenesis of FSHD and underscore the potential therapeutic implications of targeting the immune response in this condition. Besides, our findings emphasize the clinical relevance of MRI-guided biopsies for characterizing disease activity and validate TIRM hyperintensity as imaging biomarker of active disease, where activity may stand for ongoing inflammation in a dynamic and independent fashion from muscle fatty infiltration. Future studies are needed to elucidate the mechanisms underlying inflammation in FSHD, explore the role of inflammatory mediators in disease progression, and evaluate the efficacy of anti-inflammatory interventions in mitigating muscle pathology and improving clinical outcomes in FSHD patients. By better understanding the inflammatory component of FSHD, we may uncover novel therapeutic targets and strategies to address this debilitating muscle disorder.

## Supplemental Material

sj-xlsx-1-jnd-10.1177_22143602251380426 - Supplemental material for Immunohistological and electron microscopy profile of unique TIRM-MRI guided muscle biopsies of FSHD patients Supplemental material, sj-xlsx-1-jnd-10.1177_22143602251380426 for Immunohistological and electron microscopy profile of unique TIRM-MRI guided muscle biopsies of FSHD patients by Anna Greco, Benno Kusters, Ritse Mann, Jurgen Futterer, Leon de Jong, Yordy Welling, Marieke Ploegmakers, Ger JM Pruijn, Leo AB Joosten and Baziel G. M. van Engelen in Journal of Neuromuscular Diseases

sj-xlsx-2-jnd-10.1177_22143602251380426 - Supplemental material for Immunohistological and electron microscopy profile of unique TIRM-MRI guided muscle biopsies of FSHD patients Supplemental material, sj-xlsx-2-jnd-10.1177_22143602251380426 for Immunohistological and electron microscopy profile of unique TIRM-MRI guided muscle biopsies of FSHD patients by Anna Greco, Benno Kusters, Ritse Mann, Jurgen Futterer, Leon de Jong, Yordy Welling, Marieke Ploegmakers, Ger JM Pruijn, Leo AB Joosten and Baziel G. M. van Engelen in Journal of Neuromuscular Diseases

sj-xlsx-3-jnd-10.1177_22143602251380426 - Supplemental material for Immunohistological and electron microscopy profile of unique TIRM-MRI guided muscle biopsies of FSHD patients Supplemental material, sj-xlsx-3-jnd-10.1177_22143602251380426 for Immunohistological and electron microscopy profile of unique TIRM-MRI guided muscle biopsies of FSHD patients by Anna Greco, Benno Kusters, Ritse Mann, Jurgen Futterer, Leon de Jong, Yordy Welling, Marieke Ploegmakers, Ger JM Pruijn, Leo AB Joosten and Baziel G. M. van Engelen in Journal of Neuromuscular Diseases
